# “Lived experience” events by people with dementia positively influence perceptions and future care approaches

**DOI:** 10.1002/alz70858_103863

**Published:** 2025-12-26

**Authors:** Stephani Shivers, Olivia Cohen, Anne Kenny, Vicki Zimmer, Amy Puca, Edward Cisek

**Affiliations:** ^1^ CaringKind, New York, NY, USA; ^2^ Via Evaluation, Buffalo, NY, USA

## Abstract

Stigma affects how people living with dementia (PLWD) are treated/cared for and stymies early/timely diagnoses and services. PLWD can combat stigma by sharing insight into their experiences and feedback regarding the services they receive. Leveraging funding from an Administrative on Community Living – Alzheimer's Disease Program Initiatives grant, CaringKind, a dementia focused community‐based organization, launched two distinct but related programs aiming to change attitudes and behaviors towards PLWD. Small facilitated groups of PLWD crafted scripts about their experiences over 8‐10 sessions. Group members then read the script before an audience in a one‐hour performance with a question/answer period. “To Whom I May Concern®” (TWIMC) is for general audiences, while “Dementia Act II” (DA2) is designed for healthcare, researcher, and industry professionals/students. Pre‐/Post performances, audiences are asked to write three words to describe the experience of a person with “dementia”. DA2 audiences are also asked post‐performance reflective questions about their past/anticipated future interactions with PLWD around 3 themes: speaking directly to the PLWD; involvement in peer support/meaningful engagement; and connecting caring family/friends to ongoing supports. Pre‐/Post response changes have been analyzed for four events each. For TWIMC audiences, the most common descriptive words changed from “sad”, “confusion”, and “frustration” to “hope”, “love”, and “brave”. For DA2 audiences, the most common descriptive words changed from “frustration”, “memory”, “confusion” and “sad” to “hope”, “resilient”, “creative” and “strong”. Moreover, the DA2 respondents who “speak to the PLWD directly…” most or all the time rose from 78% to 95%. Similarly, DA2 respondents who “considered the PLWD's involvement in peer support and meaningful engagement…” most or all the time rose from 47% to 95%. Finally, the percentage of DA2 respondents who “consider the connectivity of those family/friends providing care to ongoing support, education, and planning services as care needs change” most or all the time rose from 51% to 94%. These results demonstrate that a one‐hour lived experience performance can improve attitudes towards PLWD. Moreover, by hearing about PLWD experiences in their own words, practicing professionals may improve how they interact with, and plan for the support and care of, families affected by dementia.

